# Empagliflozin alone and in combination with metformin mitigates diabetes-associated renal complications

**DOI:** 10.25122/jml-2023-0301

**Published:** 2024-05

**Authors:** Zena Madhag, Zahraa Al-Isawi

**Affiliations:** 1 Department of Pharmacology and Toxicology, Faculty of Pharmacy, University of Kufa, Kufa, Iraq

**Keywords:** diabetes, nephropathy, empagliflozin, metformin, inflammation, apoptosis

## Abstract

Diabetes mellitus is a major public health concern, often leading to undiagnosed micro- and macrovascular complications, even in patients with controlled blood glucose levels. Recent evidence suggests that empagliflozin and metformin have renoprotective effects in addition to their hypoglycemic action. This study investigated the potential protective effect of empagliflozin and metformin on diabetic renal complications. Forty-two adult male Sprague Dawley rats were randomized into six groups: normal control, diabetic control, metformin (250 mg/kg), empagliflozin (10 mg/kg), and combination therapy groups. Type 2 diabetes was induced in rats by a single intraperitoneal injection of streptozotocin (40 mg/kg) following two weeks of 10% fructose solution in their drinking water. Blood glucose, creatinine, urea nitrogen, inflammatory markers (IL-6, TNF-α), and renal tissue caspase-3 were assessed after eight weeks. Blood glucose, urea, creatinine, serum IL-6, TNF-α, and tissue caspase-3 were significantly decreased in the treatment groups compared to the diabetic group. The histopathological findings revealed that treatment with empagliflozin and/or metformin improved the damage in the renal tissue caused by diabetes-induced nephropathy. Moreover, co-administration of empagliflozin and metformin resulted in even better outcomes. Our data revealed that empagliflozin and metformin could improve renal function and decrease inflammation and apoptosis in diabetic animals, delaying the progression of diabetic nephropathy. Combined treatment with metformin and empagliflozin proved to have an additive protective action on renal tissue.

## INTRODUCTION

Type 2 diabetes mellitus (T2DM) is the most common form of diabetes, affecting approximately 90% of diagnosed adults [1,2]. This metabolic disorder is characterized by insulin resistance and impaired insulin secretion, often co-occurring with other health conditions. The global prevalence of diabetes among adults aged 20-79 is projected to rise from 10.5% (536.6 million) in 2021 to 12.2% (783.2 million) by 2045. This increase in prevalence is accompanied by a substantial economic burden, with the global cost of diabetes-related illnesses estimated to reach $1,054 billion by 2045 [3]. Considerable evidence suggests that T2DM can be prevented or at least postponed during the prediabetes stage through several measures. Lifestyle modifications, such as dietary changes, increased physical activity, and weight loss, have been shown to reduce the incidence of T2DM by 39-71%. Medications such as metformin can reduce it by 28%-79%, whereas metabolic surgery like gastric bypass can reduce it by 75% [4]. Early and effective management of diagnosed diabetes through diet and lifestyle modifications, including weight loss and antidiabetic medications, can significantly improve the prognosis [5-6]. Apart from hyperglycemia, diabetes causes micro and macro-vascular complications, increasing the risk of cardiovascular disease among patients with diabetes [7]. One of the most common long-term disease complications is diabetic nephropathy (DN), which occurs in 40% of patients. DN is considered the leading cause of end-stage renal disease and is associated with high mortality and morbidity among patients with chronic kidney disease [8,9]. Several strategies have been proposed to mitigate diabetic renal complications, especially those interfering with the inflammatory response, oxidative stress, and apoptotic pathways. Empagliflozin is an antidiabetic agent, one of the sodium-glucose co-transporters-2 (SGLT2) inhibitors with remarkable anti-inflammatory, antioxidant effects and antiapoptotic action [10]. Experimental studies showed that empagliflozin ameliorates symptoms of diabetes and renal tubular dysfunction in diabetic animals with enlarged kidneys [11]. Metformin, a biguanide antidiabetic agent, works independently of insulin secretion by primarily increasing insulin sensitivity in muscles, the liver, and the gastrointestinal tract (GIT). Furthermore, metformin modulates the meta-inflammation often associated with obesity, exerting both direct and indirect effects on immune cells within metabolic organs such as the liver, adipose tissue, and GIT [12]. Metformin has a nephroprotective effect by reducing apoptosis and increasing glucose uptake in patients and animals with diabetes [13]. In this study, we investigated whether empagliflozin, alone or in combination with metformin, could improve diabetic nephropathy. We hypothesized that this potential therapeutic effect would be mediated by suppressing renal inflammation and apoptosis.

## MATERIAL AND METHODS

### Study design

This study was conducted at the animal house facilities at the Faculty of Science, Kufa University, from December/2022 through March/2023. The experimental study lasted for 12 weeks. Forty-two adult male Sprague Dawley rats weighing 180-200g were housed under controlled conditions (24±2°C, 12-hour light/dark cycle) and acclimatized for two weeks. Due to higher resistance to diabetic nephropathy (DN) in females [14], only males were used. To induce diabetes (*n* = 35), rats received 10% fructose in drinking water for two weeks, followed by a single intraperitoneal injection (i.p.) of streptozotocin (STZ, 40mg/kg) [15]. Blood glucose level was measured after 72 hours using a glucometer. Animals with blood glucose levels ≥250 mg/dl were considered diabetic. After confirming experimental diabetes, they were further divided into six groups (*n* = 7 per group):

1. Normal control (NC) (*n* = 7): Standard chow diet and water *ad libitum* throughout the study.
2. Diabetic control (DM): No treatment.
3. Vehicle control (DM + vehicle): Dimethyl sulfoxide (DMSO) buffer as empagliflozin vehicle via oral gavage for eight weeks.
4. Empagliflozin (DM + EMPA): Empagliflozin (10mg/kg/day) via oral gavage for eight weeks [16].
5. Metformin (DM + Met): Metformin (250mg/kg/day) via oral gavage for eight weeks [17].
6. Combination (DM + EMPA + Met): Empagliflozin (10mg/kg/day) and metformin (250mg/kg/day) for eight weeks.

### Chemicals

Fructose powder of 99% purity (cat#8100), Streptozotocin (STZ; Cat. No. S8050), empagliflozin (Cat. No. E2280), and metformin (Cat. No. D9351) were obtained from Solarbio. The urea spectrophotometric kit (REF 1123005) and creatinine spectrophotometric kit (REF 1156010) were purchased from LiNER Cromatest. Rat interleukin-6 ELISA kit (Cat. No. E0135Ra) and Rat tumor necrosis-alpha ELISA kit (Cat. No. E0764Ra) were purchased from BT LAB. Rat CASP3 (caspase 3) ELISA kit (Cat: ELK1528) was purchased from ELK Biotechnology. Other reagents and chemicals used in this study were of analytical grade.

### Serum preparation for biochemical assays

After 12 weeks, rats were euthanized with ketamine/xylazine (75/5 mg/kg i.p.). The blood samples were collected directly by heart puncture with a 5 ml syringe. Blood was collected in a gel tube at room temperature without anticoagulant. Finally, the serum was separated by centrifugation for 15 minutes at 3000 rpm [18]. Serum obtained from blood samples was aliquoted into Eppendorf tubes for further analysis of serum urea, creatinine, tumor necrosis factor-alpha (TNF-α), and interleukin-6 (IL-6).

### Tissue preparation for caspase 3 measurement

The right kidney of each animal was immediately excised through a midline abdominal incision. After that, it was rinsed with ice-cold PBS to remove any red blood cells or clots. Tissues were minced into small pieces and homogenized in fresh cold PBS buffer (w: v = 1:9, e.g., 900 µL PBS buffer was added to 100 mg tissue sample) with a glass homogenizer on ice. Then, the homogenates were centrifuged for 5 minutes at 10000 × g to collect the supernatant. Caspase 3 was measured in the resultant supernatant using the ELISA technique.

### Biochemical analysis

Blood glucose level was determined using a Glucometer (ACCU-CHEK Active (Roche Diagnostics). Serum urea and creatinine levels were detected using the spectrophotometric kit (LiNER Cromatest). Serum tumor necrosis factor-alpha (TNF-α) and interleukin-6 (IL-6) were analyzed using the commercially available ELISA kit (BT LAB). Estimation of caspase 3 level in kidney homogenates was also performed using the ELISA kit (ELK Biotechnology). All measurements were performed according to the manufacturer’s instructions.

### Histological examination

The left kidneys of animals were fixed in 10% neutral buffered formalin and embedded in a paraffin block after dehydration by graded ethanol. Blocks were sectioned into 5-µm thicknesses and mounted on glass slides, and routine histopathological steps were followed [19]. The slides were stained with hematoxylin and eosin (H&E) to examine the histological changes by an experienced pathologist blinded to the treatment groups. Images were captured using a light microscope and digital camera at a magnification of 10X.

### Data analysis

Analyses were performed using GraphPad Prism 8 (GraphPad Software). The data were shown as mean ± standard error of the mean (SEM). Statistical significance between different treatment groups was estimated using a one-way analysis of variance (ANOVA), with Tukey's multiple comparisons as a post-analysis test. Statistical significance was set at a *P* value less than 0.05.

## RESULTS

### Empagliflozin and metformin improved blood glucose levels and renal function markers in diabetic rats

Following diabetes induction, rats had significant elevations in blood glucose, urea, and creatinine, indicating impaired glucose control and renal function compared to healthy controls. However, the administration of empagliflozin or metformin for 8 weeks revealed a significant decrease in blood glucose, urea, and creatinine levels compared to the diabetic control and vehicle groups. Moreover, combination therapy with both drugs significantly improved renal dysfunction compared with either single-drug treatment (Table 1).

**Table 1:** The effect of empagliflozin and metformin on blood glucose and renal function parameters across all groups

Parameter	Study groups					
	NC	DM	DM+Vehicle	DM+Met	DM+EMPA	DM+EMPA+Met
Blood glucose (mg/dl)	87.29±1.539	475±15.15*	482±9.008*	280±3.415^#$^	253.9±1.908^#^	240±3.922^#^
Serum urea (mg/dl)	24.43±1.238	81.44±1.379*	79.29±1.678*	51.70±0.9431^#$^	43.64±1.253^#$^	34.54±1.212^#^
Serum creatinine (mg/dl)	0.3457±0.03683	2.194±0.04455*	2.204±0.07628*	1.050±0.04943^#$^	0.7886±0.03341^#$^	0.4614±0.03341^#^

Data are presented as mean ± SEM. Normal control (NC), Diabetic control (DM), Diabetic treated with vehicle (DM + Vehicle), Diabetic treated with metformin (DM + Met), Diabetic treated with empagliflozin (DM+EMPA), Diabetic treated with empagliflozin and metformin (DM+ EMPA + Met). *P ≤ 0.05 versus normal control. #P ≤ 0.05 versus diabetic control. $P ≤ 0.05 versus combined treatment.

### Empagliflozin and metformin attenuated the inflammatory response in diabetic rats

**Figure 1:**
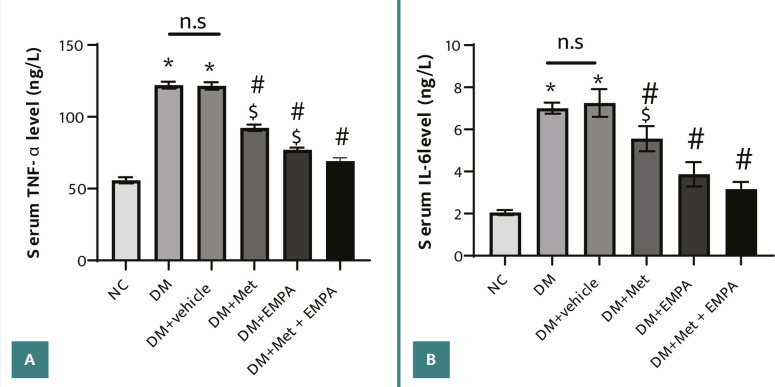
Modulatory effect of empagliflozin and/or metformin treatment on the systemic inflammatory status in diabetic rats. A, Serum TNF-α level; B, Serum IL-6 level. STZ-induced diabetic animals were treated with empagliflozin (10mg/kg/day) and/or metformin (250mg/kg/day) for eight weeks. Results are expressed as mean ± SEM (*n* = 7 rats/group), *P ≤ 0.05 versus normal control. ^#^P ≤ 0.05 versus diabetic control. ^$^P ≤ 0.05 versus combined treatment.

Diabetes significantly increased serum levels of TNF-α and IL-6 in diabetic control rats and vehicle-treated rats compared to normal controls. Nevertheless, treatment with empagliflozin and metformin significantly reduced inflammatory markers, namely TNF-α and IL-6, compared to diabetic control and vehicle groups. Higher improvement was obtained when both drugs were co-administered, as shown in Figure 1AB.

### Empagliflozin and metformin treatments combated apoptosis in the renal tissue of diabetic rats

**Figure 2:**
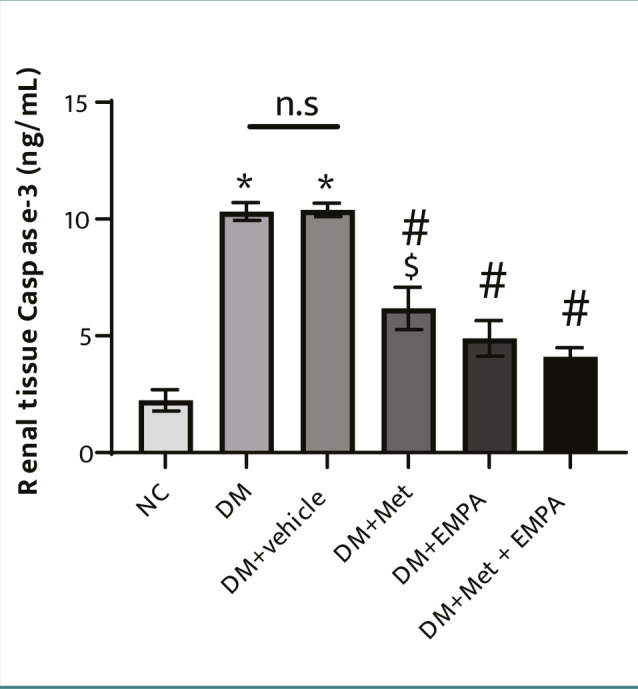
Suppressive effect of empagliflozin and/or metformin treatment on renal caspase-3 level of diabetic rats. STZ-induced diabetic animals were treated with empagliflozin (10mg/kg/day) and/or metformin (250mg/kg/day) for eight weeks. Results are expressed as mean ± SEM (*n* = 7 rats/group), *P ≤ 0.05 versus normal control. ^#^P ≤ 0.05 versus diabetic control. ^$^P ≤ 0.05 versus combined treatment.

As shown in Figure 2, caspase 3 levels, a marker of apoptosis, were significantly elevated in the kidneys of untreated diabetic rats compared to normal controls. Both empagliflozin and metformin treatment significantly reduced renal caspase 3 levels, indicating a protective effect against apoptosis. Furthermore, the combined administration of both drugs resulted in a more substantial reduction in caspase 3 levels compared to metformin alone.

### Empagliflozin and/or metformin treatments ameliorated kidney tissue injury induced by diabetic conditions

**Figure 3:**
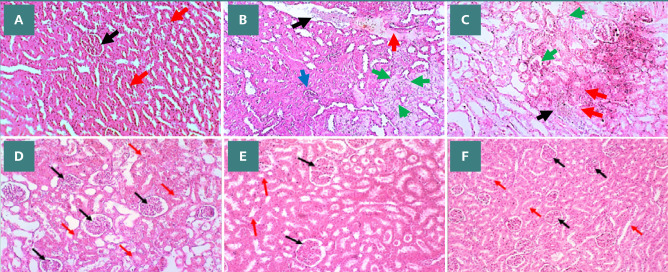
Histological analysis of kidney sections in different experimental groups. A, NC: normal renal histology; B, DM: Renal vein congestion (black arrow), severe proximal tubule damage (coagulative necrosis, red arrows), atrophic glomeruli (blue arrow), and necrotic lesions (liquefactive necrosis, green arrows); C, DM + Vehicle: Structural lesions similar to the DM group; D, DM + Met: Near-normal glomerular tuft and capsule (black arrows), moderate hypertrophy in proximal tubules (red arrows); E, DM + EMPA: Normal glomerular tuft and capsule (black arrows), mild hypertrophy in proximal tubules (red arrows); F, DM + Met + EMPA: Significant improvement with nearly normal kidney histology, showing normal glomerular tuft and proximal tubules (black and red arrows). Images were captured using a light microscope and digital camera at a 10X magnification.

Kidney sections from all the study groups were analyzed using H&E staining to evaluate the effect of diabetes on renal tissue structure and the potential modulatory effects of empagliflozin and metformin. The normal control group had normal kidney structure, showing intact glomeruli and tubules (Figure 3A). In contrast, the DM and DM + vehicle groups had multiple lesions due to chronic damage induced by diabetes in these tissues (Figure 3BC). Metformin treatment mitigated the severity of these diabetic-induced lesions, particularly in the glomeruli, with largely preserved glomerular structure and reduced tubular damage (Figure 3D). Consistently, renal tissue sections showed near normal glomerulus structures with mild hypertrophied changes in the epithelial cells of the proximal tubules lining layer in empagliflozin-treated diabetic rats (Figure 3E). The combination therapy of empagliflozin and metformin demonstrated the most significant renoprotective effects. Renal tissues in this group exhibited a near-normal appearance, with minimal evidence of glomerular or tubular damage, as shown in Figure 3F.

## DISCUSSION

Diabetes is a widely spread chronic condition that represents a serious burden to public health [3]. Although hyperglycemia is the primary acute manifestation, chronic complications, particularly diabetic nephropathy, remain a major challenge in disease management [9]. SGLT2 inhibitors have demonstrated profound renoprotective properties [11]. Therefore, the present study investigated the effects of empagliflozin alone or co-administered with metformin on renal function and histopathology in a T2DM rat model and its relationship with inflammatory and apoptotic signaling. In this study, T2DM was generated experimentally in rats by STZ and fructose administration [14]. Previous studies found that fructose is a lipogenic agent that produces insulin resistance in many organs, especially the liver [20]. STZ is a naturally occurring antibiotic produced by *Streptomyces achromogenes* bacteria. Because of its diabetogenic effects, STZ is mainly used to induce diabetes in animal models [21]. Multiple therapeutic agents have been proposed to stop the development of diabetic kidney disease, particularly those manipulating the inflammatory status, oxidative load, or apoptotic pathways [13-15]. Empagliflozin interferes with glucose reabsorption in the kidney without affecting insulin release or sensitivity. Several studies suggested a protective effect of empagliflozin on the vascular system by diminishing inflammatory and oxidative signals [22]. On the other hand, metformin demonstrated renoprotective properties in animal models of T2DM by lowering blood glucose levels and improving kidney function in addition to their anti-inflammatory and anti-apoptotic efficacy [12]. Hyperglycemia causes deleterious changes to kidney parenchyma, leading to a decline in renal function. Prolonged elevated blood glucose levels in untreated diabetic animals result in increased urea and creatinine levels, indicative of impaired kidney function. These deleterious effects are attributed to damage to essential renal structures like the glomerular basement membrane and podocytes, which are crucial for maintaining normal filtration and preventing protein loss in urine [23]. Experimental studies reported that treatment with metformin or empagliflozin recovers the functional capacity of the diabetic kidney and improves the histopathological picture in the renal tissue in animal models of diabetes with renal involvement [11,15]. Diabetes is an inflammatory disease, and elevated blood glucose level and insulin insensitivity leads to the activation of immune cells, thereby secreting interleukins and cytokines such as IL-6 and TNF-α and aggravating the inflammatory response [23-25]. Clinical studies have found that TNF-α is significantly increased in patients with diabetes. Moreover, this cytokine plays a pivotal role in the development of diabetes microvascular complications, including DN. TNF-α has cytotoxic effects on the mesangial, epithelial, and glomerular cells of the kidney, contributing to renal injury [26]. Empagliflozin, recognized for its pleiotropic actions, has demonstrated anti-inflammatory, antioxidant, and anti-apoptotic effects across multiple disease models [10-11, 25]. These beneficial effects are attributed to its ability to inhibit downstream effectors of the pro-inflammatory transcription factor nuclear factor kappa B (NF-κB), thereby suppressing the inflammatory cascade [27]. In line with these findings, metformin treatment also demonstrated anti-inflammatory effects, reducing systemic cytokine release and interleukin levels and exerting a nephroprotective action in diabetic as well as non-diabetic animal models of chronic kidney disease [17]. Numerous studies have highlighted the pivotal role of hyperglycemia in promoting oxidative stress and inflammation, ultimately leading to the activation of apoptotic pathways within renal tissue [16]. This diabetic milieu triggers programmed cell death, as evidenced by elevated levels of the apoptotic marker caspase 3, which further exacerbates renal injury, including glomerular and tubular damage [28]. Empagliflozin has been shown to mitigate renal tubular apoptosis by reducing caspase-3 protein levels in streptozotocin-induced diabetic rats [27]. Similarly, metformin exerts anti-apoptotic effects by downregulating Bax, caspase-3, and caspase-9 activity in diabetic animals [12]. This anti-apoptotic mechanism is mediated through the activation of AMP-activated protein kinase (AMPK) and subsequent regulation of the AMPK/SIRT1-FoxO1 pathway, which plays a crucial role in autophagy and cellular homeostasis in diabetic nephropathy [16]. In diabetic animals, elevated glucose levels induce various changes in renal histology. Animal models of T2DM have been shown to successfully replicate the renal histopathological injury observed in diabetic patients with kidney involvement [29]. The extent of structural damage correlates with disease duration and the degree of glycemic control [6-9]. Early histological changes include glomerular basement membrane thickening, renal tubule dilation or atrophy, and glomerular hypertrophy. In later stages, more severe lesions such as glomerulosclerosis and interstitial fibrosis may develop [27]. The pathogenesis of diabetic kidney disease has been conventionally attributed to hemodynamic alterations and severe persistent hyperglycemia. However, accumulating evidence highlights the vital contribution of inflammation and oxidative load to the development and progression of kidney disease [16]. In the diabetic kidney, infiltration of innate immune cells and a surge in systemic inflammatory cytokines and chemokines contribute to cellular injury [23]. Previous studies have shown that empagliflozin and metformin have nephroprotective properties, improving the structural alterations in the renal tissue triggered in diabetic and nondiabetic settings [12,30]. Empagliflozin reduces renal and tubular injury by mitigating the renal inflammatory process, injury, and damage [31]. Additionally, a previous investigation found that treatment with metformin dramatically reduced the pathogenic aspects of T2DM by lowering blood sugar, preserving renal functions, and maintaining normal morphology. The mechanism causing this effect may be linked to lipid metabolism, glycemic management, and anti-oxidative and anti-inflammatory processes [32].

## CONCLUSION

This study suggests that treatment with empagliflozin and metformin in diabetic conditions effectively reduces inflammation and apoptosis and improves renal function. Empagliflozin and metformin ameliorated diabetic nephropathy in diabetic rats. However, combining them resulted in a further reduction in kidney injury via anti-inflammatory and anti-apoptotic pathways.
